# Heat-killed *Bacillus subtilis* concerning broilers’ performance, cecal architecture and microbiota

**DOI:** 10.3389/fmicb.2025.1606352

**Published:** 2025-06-27

**Authors:** Yan Cui, Weishuang Meng, Feng He, Zeliang Chen, Hongliang Liu, Desheng Li

**Affiliations:** ^1^College of Husbandry and Veterinary Medicine, Jinzhou Medical University, Jinzhou, China; ^2^Key Laboratory of Animal Product Quality and Safety of Liaoning Province, Jinzhou Medical University, Jinzhou, China; ^3^Liaoning Kaiwei Biotechnology Co., Ltd., Jinzhou, China; ^4^Collaborative Innovation Center for Prevention and Control of Zoonoses, Jinzhou Medical University, Jinzhou, China; ^5^Jiangsu Yinong Biological Co., Ltd., Jiangsu, China

**Keywords:** additive, hindgut, intestinal health, intestinal fungus composition, ITS, postbiotic

## Abstract

**Background:**

At present, *Bacillus subtilis* has been reported as a probiotic to be used as a feed additive to improve the production performance of broilers. However, the relevant reports on its derived postbiotics are still limited.

**Methods:**

In this study, a total of 480 day-old Arbor acre broiler chicks with an initial body weight of 52.8 ± 1.4 g were used in this study to investigate the effects of dietary supplementation of graded levels of heat-killed *B. subtilis* on growth performance, cecal morphology, and cecal bacteria and fungus composition. Birds were assigned into four groups with six replicates of 20 birds each. The experiment was designed to last for 42 days. *B. subtilis* was supplemented with 0.000, 0.015, 0.030, or 0.045% heat-killed *Bacillus subtilis* (HKB).

**Results:**

Our data indicate that body weight gain, gain to feed ratio, cecal villus height, and cecal villus to crypt ratio dose-dependently improved, while cecal crypt depth dose-dependently reduced, by HKB supplementation. Additionally, dietary supplementation of graded levels of HKB dose-dependently reduced cecal bacteria Chao1 index. Cecal bacteria identified by 16S rRNA and cecal fungus identified by ITS revealed that the supplementation of HKB regulate the composition of bacteria and fungus in cecum.

**Conclusion:**

Therefore, dietary supplementation of HKB is an effective measure to improve cecal morphology by regulating cecal bacteria and fungus composition, and therefore improved growth performance.

## Introduction

The poultry industry has historically relied on antibiotic supplementation to bolster growth performance and intestinal health, a practice that has yielded significant benefits (Mehdi et al., 2018). However, the recognition of the adverse effects linked to antibiotic use has led to a shift in focus toward antibiotic-free poultry farming (Muhammad et al., 2020). In this transition, the industry has encountered challenges marked by suboptimal growth (Cervantes, 2015). The intestinal well-being of broilers is pivotal for their growth and development, making it a critical area of focus in modern poultry management practices.

Broilers are monogastric animals, and the health of their hindgut significantly influences their growth performance. The hindgut is mainly responsible for fermenting the undigested nutrients in the small intestine through microorganisms into nutrients that could be absorbed by the body (such as short-chain fatty acids), which are then absorbed and utilized by the body (Qaisrani et al., 2014). Therefore, the gut microbiota in the hindgut contribute significantly to the digestion process and influence feed utilization. A balanced and healthy hindgut microbial community enhances nutrient breakdown and absorption, positively affecting both intestinal morphology and overall growth performance (Rinttila and Apajalahti, 2013). Studies, such as those conducted by Mohamed et al. (2021) and Oladokun et al. (2021) have demonstrated that strategies aimed at improving cecal morphology and the composition of cecal microbiota are effective measures to enhance growth performance in broilers. Therefore, it is crucial to implement targeted interventions to enhance the hindgut health of broilers, ensuring favorable growth outcomes.

Probiotic supplementation has emerged as an effective strategy, demonstrated by its ability to maintain intestinal health (Khan et al., 2020) and enhance growth performance (Dang et al., 2024). *Bacillus subtilis*, in particular, has been extensively studied for its positive effects on intestinal health (Zhang et al., 2021). Al-Fataftah and Abdelqader (2014) reported that the inclusion of *B. subtilis* in the diet of heat-stressed broilers was partially effective in overcoming the adverse effects on performance by restoring the impaired villus-crypt structure and enhancing the colonization of beneficial intestinal bacteria. Additionally, Oladokun et al. (2021) demonstrated that in ovo delivery of *B. subtilis* enhanced intestinal morphology of broilers. Furthermore, Jacquier et al. (2019) found that dietary supplementation of *B. subtilis* 29784 significantly improved the growth performance of broilers, likely due to its beneficial effects on the intestinal microbiota.

It is crucial to highlight that the beneficial effects of probiotics are not solely contingent upon their viability (Kataria et al., 2009). Research has shown that non-viable microorganisms, including inactive bacteria, can also confer advantageous outcomes in terms of poultry growth and production (Nofouzi et al., 2021). These non-viable microorganisms are commonly referred to as “postbiotics.” In alignment with the concept of postbiotics, as elucidated by the International Scientific Association of Probiotics and Prebiotics in 2021, a postbiotic is defined as a “preparation of inanimate microorganisms and/or their components that bestow a health benefit upon the host” (Salminen et al., 2021). Although research on *B. subtilis*-derived postbiotics in improving hindgut health is still limited, promising findings have been reported. Zhu et al. (2020) noted that dietary supplementation with heat-inactivated *B. subtilis* and *Lactobacillus acidophilus* improved animal growth performance by influencing intestinal microbiota.

Therefore, a postbiotic derived from *B. subtilis* may serve as an effective additive to improve the growth performance of broilers by regulating hindgut morphology and microbiota composition. It’s worth noting that the interplay between intestinal bacteria and fungi is a vital aspect often overlooked (Perez and Johnson, 2013). In gnotobiotic mice, a small community of fungi was found to induce significant changes in gut bacterial assembly (van Tilburg et al., 2020). Understanding the interaction of fungi and bacteria within the gastrointestinal tract is crucial for comprehensively assessing gut health. Therefore, in this study, broilers were chosen as the experimental animals to explore the impact of *B. subtilis*-derived postbiotic supplementation on the composition of cecal microbiota using 16S rRNA and ITS sequencing techniques, along with the analysis of cecal morphology and growth performance. We hypothesized that killed *B. subtilis* intake would improve cecal morphology by modulating the composition of cecal bacteria and fungi, thereby enhancing overall growth performance.

## Materials and methods

### Experimental design

A total of 480 day-old Arbor acre broiler chicks with a similar initial body weight of 52.8 ± 1.4 g were used in this study. Birds were randomly assigned into four groups based on their initial body weight. Birds were assigned into four groups with six replicates of 20 birds each. The experiment was designed to last for 42 days. *B. subtilis* was supplemented with 0.000, 0.015, 0.030, or 0.045% heat-killed *Bacillus subtilis* (HKB). The HKB utilized in this study was sourced from *B. subtilis* ACCC 11025 and obtained from Jiangsu Yinong Biological Co., Ltd. (Jiangsu, China). The diet formulation provided to the birds was optimized based on the National Research Council. These formulations were successfully applied in commercial settings by Boin Feed Company, Shenyang, China (Supplementary Table 1). The experimental protocol and all procedures were meticulously conducted in accordance with stringent ethical standards. The study was approved and supervised by the Animal Care and Use Committee of Jinzhou Medical University (Jinzhou, China), ensuring the welfare and ethical treatment of the animals involved in the research (protocol number JAU20250120).

All the birds were housed in three-tier battery cages, each measuring 1.25 meters in length, 0.80 meters in width, and 0.50 meters in height. These cages were placed in a controlled experimental environment where temperature conditions were carefully regulated. The temperature was initially set at 33°C and gradually reduced by 3°C every week until it reached 22°C. The relative humidity was maintained at 65%. Throughout the entire duration of the experiment, the birds had unrestricted access to both feed and water. The nutritional requirements of feed formula are optimized from the nutritional requirements recommended by the National Research Council and successfully applied in commercial (Boin feed company, Shenyang, China) (Supplementary Table 1). Drinking water is clean groundwater. During the whole feeding process, incandescent lamps were used for illumination. Light 22 h a day in the first week and the last week, light intensity 10 lux; in the second and fifth weeks, 20 h of light per day, light intensity 5 lux; in the third and fourth weeks, 18 h of light per day, light intensity 5 lux. Seven days before the chicks are introduced, the chicken coop should be thoroughly disinfected with sodium hypochlorite. Negative pressure ventilation was used throughout the test period.

### Parameters measurement

#### Growth performance

The body weight of broilers was measured on both the initial and final day of the study to calculate the body weight gain (BWG) (Luo et al., 2022). Furthermore, the cage-based feed intake (FI) was measured weekly to calculate the FI of the broilers. Additionally, feed efficiency (gain-to-feed ratio) was computed using the data from BWG and FI.

#### Cecal morphology

After 12 h fasting on the final day, 18 birds were randomly selected, with three birds chosen from each replicate cage, and were euthanized humanely by administering 1 mL of sodium pentobarbital intravenously (Luo et al., 2022). The cecal tissue was carefully removed from the carcasses, and small pieces of cecal samples were prepared for paraffin blocks following the protocol described by Dang et al. (2023). Tissue sections with a thickness of 5 μm were obtained using a cryostat. After sectioning, the samples were stained using the hematoxylin and eosin staining protocol as outlined by Felicio et al. (2013). Subsequently, the samples were dehydrated and mounted. In each specimen, the villus height and crypt depth were meticulously measured under a light microscope equipped with a ScopePhpto (LY-WN 300, Hangzhou Scopetek Opto-Eletric Co., Ltd.). A minimum of five measurements per slide were taken for each parameter and were averaged to obtain a single value. The values presented in the study are the means derived from measuring 10 adjacent villi, and only vertically oriented villi were included in the measurements.

#### Cecal microbiota

Cecal contents obtained from the euthanized animals were processed for DNA extraction utilizing a magnetic Soil and Stool DNA kit (cat# DP712, TIANGEN Biotech Co., Ltd., Beijing, China). The concentration and purity of the extracted DNA were assessed using a Qubit 2.0 spectrophotometer (Invitrogen, Carlsbad, CA, USA) and 1% (w/v) agarose gel electrophoresis. Subsequently, the DNA samples were diluted to a concentration of 1 ng/μL with sterile water and stored at −20°C until analysis. For microbial community analysis, the V3–V4 hypervariable regions of the bacterial 16S rRNA gene were amplified using specific full-length universal forward (5′-ACTCCTACGGGAGGCAGCA-3′) and reverse (5′-GGACTACHVGGGTWTCTAAT-3′) primers. The fungal ITS region (V1) was amplified using specific full-length universal forward (5′-GGAAGTAAAAGTCGTAACAAGG-3′) and reverse (5′-GCTGCGTTCTTCATCGATGC-3′) primers. The PCR reactions were carried out in triplicate, and the resulting PCR products were purified using a Qiagen Gel Extraction Kit (cat# 28706, Qiagen, Germany). The purity of the PCR mixture was confirmed using a Qubit 2.0 dsDNA HS Assay Kit (cat# Q32854, Invitrogen). Microbial community structures were analyzed through 16S rRNA sequencing and ITS sequencing, conducted on the NovaSeq 6000 platform (Illumina, San Diego, CA, USA) at Novogene Bioinformatics Co., Ltd. (Tianjin, China).

The raw sequencing data underwent rigorous processing steps to ensure accuracy and reliability. Initially, low-quality reads were eliminated using Cutadapt software version 1.9.1, and chimeric sequences were trimmed using alignment and detection processes. The remaining high-quality reads were clustered into operational taxonomic units (OTUs) at 97% sequence identity using Uparse v7.0.1001. Taxonomic assignment of representative sequences was conducted using QIIME v1.9.1. To assess microbial diversity, rarefaction curves were generated for each sample in R software (version 1.9.1) to determine the appropriate sequencing depth, capturing the extent of microbial diversity. Various alpha-diversity metrics, including Chao1, Pielou_e, Shannon, and Simpson diversity indices, were calculated based on the observed OTUs. Additionally, beta-diversity analysis was performed using the Bray Curtis distance metric.

### Statistical analysis

All collected data underwent normality testing using the Shapiro–Wilk test and quantile–quantile plots to ensure adherence to the normal distribution. Each replicate was treated as an experimental unit for analysis. The data were then subjected to statistical analysis in a randomized complete block design utilizing the General Linear Models procedures (SAS Institute, Cary, NC, USA). The statistics were analyzed by one-way analysis of variance (one-way ANOVA). Orthogonal contrasts were employed to examine the linear and quadratic effects in response to the increasing dietary supplementation of HKB. Statistical methods such as Permanova, Anosim, Permdisp, and Adonis were applied to evaluate the beta-diversity of intestinal bacteria and fungi. Furthermore, differences among groups were assessed using Tukey post-hoc tests for multiple comparisons. The variability in the data was expressed as the standard error of means, and a significance level of *p* < 0.05 was considered statistically significant.

## Results

Dietary supplementation of graded levels of HKB led to significant linear (*p* < 0.01) and quadratic (*p* < 0.01) increases in the body weight gain and feed efficiency of broilers. However, it did not have any significant effect on feed intake (Table 1).

**Table 1:** Growth performance of broilers as affected by supplementing graded levels of heat-killed *Bacillus subtilis*^1^.

Items	Heat-killed *Bacillus subtilis*, %				SEM^2^	*p*-value	
	0.000	0.015	0.030	0.045		Linear	Quadratic
Body weight gain, g	2,590.89^c^	2,894.40^a^	2,804.90^b^	2,844.59^ab^	24.89	<0.01	<0.01
Feed intake, g	3,815.36	3,819.80	3,820.36	3,821.65	3.31	0.20	0.64
Feed efficiency	0.68^c^	0.76^a^	0.73^b^	0.74^ab^	0.01	<0.01	<0.01

1 Values represent the means of 6 replicates per group (*n* = 6).

2 Standard error of the mean.

^a,b,c^Means in the same row with different superscript differ significantly (*p* < 0.05).

The supplementation of graded levels of HKB had a significant linear improvement on cecal villus height (*p* = 0.01), led to linear (*p* < 0.01) and quadratic (*p* < 0.01) reductions in cecal crypt depth, and resulted in linear (*p* < 0.01) and quadratic (*p* < 0.01) increases in cecal villus to crypt ratio. The post-hoc test indicated that any level of HKB supplementation produced positive effects on cecal villus height and cecal villus to crypt ratio, while it generated negative effects on cecal crypt depth (*p* < 0.05) (Table 2).

**Table 2:** Cecal morphology of broilers as affected by supplementing graded levels of heat-killed *Bacillus subtilis*^1^.

Items	Heat-killed *Bacillus subtilis*, %				SEM^2^	*p*-value	
	0.000	0.015	0.030	0.045		Linear	Quadratic
Villus height, μm	248.69^b^	312.44^a^	295.72^a^	314.60^a^	14.19	0.01	0.13
Crypt depth, μm	84.29^a^	62.42^bc^	56.24^c^	68.98^b^	3.80	<0.01	<0.01
Villus to crypt ratio	2.99^b^	5.05^a^	5.37^a^	4.58^a^	0.27	<0.01	<0.01

1 Values represent the means of 6 replicates per group (*n* = 6).

2 Standard error of the mean.

^a,b,c^Means in the same row with different superscript differ significantly (*p* < 0.05).

Nevertheless, the dietary supplementation of graded levels of HKB did not impact the alpha-diversity of cecal fungi. However, it linearly decreased the Chao1 index of cecal bacteria (*p* < 0.01). The post-hoc test indicated that HKB at dosages of 0.030 and 0.045 had negative effects on the Chao1 index of cecal bacteria (*p* < 0.05) (Table 3).

**Table 3:** Alpha-diversity of cecal bacteria and fungi as affected by supplementing graded levels of heat-killed *Bacillus subtilis*^1^.

Items	Heat-killed *Bacillus subtilis*, %				SEM^2^	*p*-value	
	0.000	0.015	0.030	0.045		Linear	Quadratic
Bacteria							
Chao1	1,469.30^a^	1,307.90^ab^	1,157.60^b^	1,205.90^b^	71.77	<0.01	0.16
Pielou_e	0.77	0.79	0.77	0.78	0.01	0.71	0.68
Shannon	8.04	8.11	7.80	7.94	0.15	0.35	0.79
Simpson	0.99	0.99	0.99	0.98	<0.01	0.47	0.72
Fungi							
Chao1	48.28	51.08	41.50	41.93	3.95	0.12	0.77
Pielou_e	0.36	0.29	0.25	0.30	0.07	0.49	0.34
Shannon	2.01	1.62	1.35	1.62	0.38	0.40	0.39
Simpson	0.62	0.51	0.47	0.55	0.11	0.62	0.40

1 Values represent the means of 6 replicates per group (*n* = 6).

2 Standard error of the mean.

^a,b^Means in the same row with different superscript differ significantly (*p* < 0.05).

There was no statistical difference observed in the beta-diversity of cecal bacteria and fungi based on Permanova, Anosim, Permdisp, and Adonis analyses (Table 4).

**Table 4:** The *p*-value of beta-diversity of cecal bacteria and fungi as affected by supplementing graded levels of heat-killed *Bacillus subtilis*^1^.

Items	Permanova	Anosim	Permdisp	Adonis
Bacteria	0.21	0.13	0.21	0.19
Fungi	0.98	0.91	0.71	0.98

1 Values represent the means of 6 replicates per group (*n* = 6).

The analysis of cecal bacteria composition using the 16S rRNA sequencing technique revealed that graded levels of HKB supplementation had specific effects. There was a linear increase in the abundance of *Blautia*, *Coprococcus*, *cc_115*, *Dehalobacterium*, *Thermoactinomyces*, *Allobaculum*, and *Lactococcus* (*p* < 0.05). Additionally, a quadratic increase was observed in the abundance of *Bilophila*, *Clostridium*, *Thermoactinomyces*, *Allobaculum*, *Lactococcus*, *Oceanobacillus*, and *Cetobacterium* (*p* < 0.05). Conversely, there was a linear decrease in the abundance of *Anaerotruncus* and *Bacillus*, and a quadratic decrease in the abundance of *Bifidobacterium* and *Bacillus* due to HKB supplementation (*p* < 0.05). The post-hoc analysis revealed distinct effects of dietary supplementation with HKB at different levels. At 0.015% HKB, there was a decrease in *Bifidobacterium* and *Bacillus* abundance (*p* < 0.05), while *Thermoactinomyces*, *Allobaculum*, *Oceanobacillus*, and *Cetobacterium* abundance increased (*p* < 0.05). Similarly, at 0.030% HKB, *Coprococcus*, *Bilophila*, *Clostridium*, *Thermoactinomyces*, *Allobaculum*, and *Lactococcus* abundance increased (*p* < 0.05), while *Bifidobacterium*, *Anaerotruncus*, and *Bacillus* abundance decreased (*p* < 0.05). Moreover, at 0.045% HKB, there was an increase in *Blautia*, *Coprococcus*, *cc_115*, *Dehalobacterium*, *Thermoactinomyces*, *Allobaculum*, and *Lactococcus* abundance (*p* < 0.05), coupled with a decrease in *Anaerotruncus* and *Bacillus* abundance (*p* < 0.05) (Table 5).

**Table 5:** Composition of cecal bacteria and fungi as affected by supplementing graded levels of heat-killed *Bacillus subtilis*^1^.

Items	Heat-killed *Bacillus subtilis*, %				SEM^2^	*p*-value	
	0.000	0.015	0.030	0.045		Linear	Quadratic
Bacteria							
*Blautia*	0.0090^b^	0.0150^ab^	0.0114^b^	0.0223^a^	0.02	0.03	0.49
*Coprococcus*	0.0081^b^	0.0126^ab^	0.0143^a^	0.0168^a^	<0.01	<0.01	0.56
*Bilophila*	0.0047^b^	0.0083^ab^	0.0113^a^	0.0056^ab^	<0.01	0.52	0.03
*cc_115*	0.0037^b^	0.0077^ab^	0.0057^b^	0.0118^a^	<0.01	0.01	0.56
*Bifidobacterium*	0.0129^a^	0.0006^b^	0.0050^b^	0.0034^ab^	<0.01	0.06	0.03
*Clostridium*	0.0019^b^	0.0027^ab^	0.0032^a^	0.0024^ab^	<0.01	0.21	0.03
*Anaerotruncus*	0.0035^a^	0.0024^ab^	0.0008^b^	0.0008^b^	<0.01	0.03	0.54
*Dehalobacterium*	0.0006^b^	0.0010^ab^	0.0007^b^	0.0015^a^	<0.01	0.04	0.39
*Thermoactinomyces*	0.0001^c^	0.0004^ab^	0.0005^a^	0.0003^b^	<0.01	0.02	<0.01
*Allobaculum*	5.95E−06^b^	0.0003^a^	0.0002^a^	0.0003^a^	<0.01	<0.01	0.03
*Lactococcus*	0^c^	0.0001^bc^	0.0005^a^	0.0002^b^	<0.01	<0.01	<0.01
*Oceanobacillus*	0^b^	0.0003^a^	0.0002^ab^	4.46E−05^b^	<0.01	0.83	<0.01
*Bacillus*	0.0002^a^	0^b^	0^b^	7.16E−05^b^	<0.01	0.04	<0.01
*Cetobacterium*	0^b^	8.40E−05^a^	4.97E−05^ab^	1.80E−05^ab^	<0.01	0.87	0.04
Fungi							
*Saccharomyces*	0.0010^b^	0.0014^a^	0.0015^a^	0.0011^ab^	<0.01	0.43	0.01
*Coniochaeta*	0.0004^a^	8.05E−05^bc^	1.41E−05^c^	0.0002^ab^	<0.01	0.15	<0.01
*Tausonia*	0.0003^a^	3.35E−05^b^	5.12E−05^b^	2.37E−05^b^	<0.01	<0.01	<0.01

1 Values represent the means of 6 replicates per group (*n* = 6).

2 Standard error of the mean.

^a,b,c^Means in the same row with different superscript differ significantly (*p* < 0.05).

Additionally, analysis of cecal fungi composition using the ITS sequencing technique revealed specific effects of graded HKB supplementation. There was a linear decrease in *Tausonia* abundance (*p* < 0.01), whereas *Saccharomyces* abundance quadratically increased (*p* = 0.01). Furthermore, a quadratic decrease was observed in both *Coniochaeta* (*p* < 0.01) and *Tausonia* (*p* < 0.01) abundance due to HKB supplementation. The post-hoc analysis demonstrated specific effects of dietary HKB supplementation at different levels on cecal fungi composition. At 0.015 and 0.030% HKB, there was an increase in *Saccharomyces* abundance, along with a decrease in *Coniochaeta* and *Tausonia* abundance (*p* < 0.05). Moreover, at 0.045% HKB, *Tausonia* abundance decreased (*p* < 0.05) (Table 5).

## Discussion

The growth performance of broilers holds immense significance in the realm of poultry farming, and achieving efficient growth remains a primary goal. In this study, the graded supplementation of HKB in the broilers’ diet exhibited positive effects on BWG, aligning with expectations and consistent with previous research findings. For instance, Incharoen et al. (2019) demonstrated that dietary supplementation with heat-killed *L. plantarum* L-137 effectively increased BWG in broilers. Similarly, Khonyoung and Yamauchi (2019) found that supplementation with heat-killed *L. sakei* HS-1 led to increased BWG in broilers. Although the bacterial source of additive in this study differed, the conclusion that dietary supplementation of HKB enhances BWG in broilers remains valid. It is essential to recognize that BWG in broilers is influenced by a myriad of factors, with efficient nutrient conversion from feed to body weight being a pivotal factor. This efficient nutrient utilization, often termed feed efficiency, plays a crucial role in determining how effectively broilers transform feed into body weight. In this study, the supplementation of HKB significantly improved the feed efficiency of broilers. In summary, the results affirm that dietary supplementation of HKB positively influences the BWG of broilers. This effect can be attributed to the enhancement of feed efficiency, showcasing the potential of HKB as a valuable dietary supplement in optimizing broiler growth performance.

The hindgut is responsible for breaking down complex nutrients that were not digested in the small intestine, allowing animals to extract the maximum nutrients from their food (Lindberg, 2023). The physiology of the intestinal tract, specifically factors like villus height, crypt depth, and their ratio, is fundamental in ensuring high feed efficiency (Jha et al., 2019). The villus to crypt ratio serves as a key indicator of the functional capacity of the intestine. An increased ratio suggests that villi, which are responsible for nutrient absorption, are longer compared to the crypts, which produce intestinal cells. This improvement indicates a larger surface area for nutrient absorption (Jha et al., 2020). In the current study, we observed that dietary supplementation with graded levels of HKB had positive effects on cecal morphology. Similar findings were reported by Incharoen et al. (2019), they observed that dietary supplementation of heat-killed *L. plantarum* L-137 positively enhanced villus height, villus area, and the ratio of villus height to crypt depth in the jejunum of broilers. Likewise, Nofouzi et al. (2021) found that dietary supplementation of heat-killed *Tsukamurella inchonensis* effectively increased intestinal crypt depth, crypt number, and goblet cell number in broilers. These findings collectively demonstrate that HKB serves as an effective additive in enhancing cecal morphology. The improvements in cecal health and morphology can contribute to more efficient nutrient absorption, leading to positive effects on overall growth performance in animals.

Additionally, the hindgut, harboring a diverse microbial population, plays a crucial role in shaping intestinal morphology (Xiong et al., 2022). It is report that dietary supplementation of probiotics improved intestinal morphology of broilers by modulating the composition of intestinal microbiota (Dang et al., 2024). In the current study, graded levels of HKB supplementation were observed to have regulatory effects on the cecal microbiota. Notably, at doses of 0.030 and 0.045% HKB, a reduced Chao1 index for cecal bacteria was noted. The Chao1 index serves as a measure of biodiversity, estimating the species richness within a microbial community. A higher Chao1 index indicates greater species richness in the intestinal microbiota (Li et al., 2022). This finding aligns with the study by Zhu et al. (2020), where supplementation of heat-inactivated *B. subtilis* and *L. acidophilus* BFI increased the beta-diversity index of cecal microbiota and the relative abundances of specific microbial taxa, while reducing the abundance of certain genera. Hence, the results from this study indicate that dietary supplementation of HKB exerts regulatory effects on the richness of cecal bacteria. However, these effects appear to vary with different dosages of HKB.

In our study, we observed that dietary supplementation of HKB at a low dosage led to a decrease in *Bifidobacterium* and *Bacillus* abundance, while increasing the abundance of *Thermoactinomyces*, *Allobaculum*, *Oceanobacillus*, and *Cetobacterium*. *Bifidobacterium* and *Cetobacterium*, key component of the gut microbiota, plays a vital role in protecting against intestinal barrier dysfunction. Studies have demonstrated its protective effects, including the inhibition of proinflammatory cytokine secretion, suppression of zonulin protein release, and improvement of intestinal tight junction integrity, all of which contribute to a healthy intestinal barrier (Bergmann et al., 2013; Chun et al., 2020; Ewaschuk et al., 2008; Lin et al., 2020). *Bacillus*, a well-known probiotic, is reported to positively influence intestinal morphology, emphasizing its importance in gut health (Al-Fataftah and Abdelqader, 2014). Furthermore, an increased relative abundance of *Thermoactinomyces* has been associated with improved intestinal characteristics, including enhanced fold height and muscular thickness, indicating better gut health (Chen et al., 2022). *Allobaculum*, known for its anti-inflammatory properties, has been linked to mitigating conditions like Ulcerative colitis, highlighting its role in intestinal health (Mo et al., 2022; Pujo et al., 2021; Vallianou et al., 2021). Similarly, *Oceanobacillus*, a probiotic, supports a healthy intestine in aquatic animals, emphasizing its beneficial impact on gut microbiota (Panigrahi et al., 2020; Busti et al., 2024; Sun et al., 2020).

When HKB was supplemented at a medium level, we observed an increase in *Coprococcus*, *Bilophila*, *Clostridium*, *Thermoactinomyces*, *Allobaculum*, and *Lactococcus* abundance, while *Bifidobacterium*, *Anaerotruncus*, and *Bacillus* abundance decreased. *Thermoactinomyces* and *Allobaculum*, both considered probiotics, have been associated with positive effects on gut health. In contrast, *Bifidobacterium* and *Bacillus* are also probiotics known for their beneficial impact on the intestinal environment. *Coprococcus* is regarded as a biomarker for a healthy intestinal barrier function, with some species known to be major producers of short-chain fatty acids, contributing to anti-inflammatory and immunity processes (Louis and Flint, 2017). Conversely, *Bilophila* has been linked to increased inflammation, intestinal barrier dysfunction, and its association with conditions like colorectal cancer (Dahmus et al., 2018; Natividad et al., 2018). *Clostridium*, another probiotic, plays an important role in various inflammatory disorders of the gut, further emphasizing its significance in gut health (Guo et al., 2020; Tuovinen et al., 2013). *Lactococcus*, also considered a probiotic, has been associated with positive effects on intestinal morphology, such as increased villus number and reduced villus width (Yao et al., 2021). *Anaerotruncus* has been related to intestinal permeability indices, and increased abundance of butyrate-producing bacteria *Anaerotruncus* has been associated with a fortified intestinal barrier by strengthening epithelial defense functions (Wang et al., 2018).

Conversely, supplementing HKB at a high dose resulted in increased abundance of *Blautia*, *Coprococcus*, *cc_115*, *Dehalobacterium*, *Thermoactinomyces*, *Allobaculum*, and *Lactococcus*, while decreasing *Anaerotruncus* and *Bacillus* abundance. As mentioned above, *Coprococcus*, *Thermoactinomyces*, *Allobaculum*, *Lactococcus*, *Anaerotruncus*, and *Bacillus* are probiotics related to promoting a healthy gut environment. Additionally, *Blautia*, recognized as a probiotic, is involved in inflammatory diseases and may have implications for immune regulation and gut health (Liu et al., 2021). The role of *cc_115* in intestinal morphology remains unclear, but studies have linked its abundance in the intestine to increased enzyme activities, improved growth performance, and enhanced antioxidant abilities (Wu et al., 2020, 2018). *Dehalobacterium*, known for its anti-intestinal inflammatory effects, has been associated with decreased intestinal permeability, indicating its potential in maintaining a healthy gut barrier (Coretti et al., 2017; Zhang et al., 2022).

The observed changes in microbiota composition highlight the potential of HKB in modulating the gut bacteria. Moreover, the supplementation of HKB also has significant effects on intestinal fungal composition, with different doses of HKB leading to distinct impacts on the fungal community. Our findings revealed that at low and medium dosages, HKB supplementation increased *Saccharomyces* abundance while decreasing *Coniochaeta* and *Tausonia* abundance. *Saccharomyces*, a well-known probiotic, exhibits beneficial activities such as modifying the intestinal microbiota, reducing the risk of dysbiosis, and producing essential vitamins and enzymes (Latorre et al., 2015). The exact functions of *Coniochaeta* and *Tausonia* in the gut remain unclear, but their presence in the intestine of broilers suggests a potential role in the complex microbial ecosystem. Notably, at a high dosage, HKB supplementation led to a decreased abundance of *Tausonia*. These results emphasize the regulatory impact of dietary HKB supplementation on the intestinal fungal composition.

It is noteworthy that dietary supplementation of HKB at any dosage affects the abundance of *Thermoactinomyces*, *Allobaculum*, *Bacillus* bacteria, and *Tausonia* fungus. These specific microbial changes indicate that *Thermoactinomyces*, *Allobaculum*, *Bacillus* bacteria, and *Tausonia* fungus might be key components influenced by HKB supplementation. However, the intricate relationship between these bacteria and fungus remains unknown. Further studies are necessary to comprehensively evaluate the complex interplay among *Thermoactinomyces*, *Allobaculum*, *Bacillus* bacteria, and *Tausonia* fungus within the intestinal environment.

In conclusion, the present study investigated the effects of dietary supplementation of HKB on the growth performance, cecal morphology, and microbiota composition in broilers. Our findings demonstrated that graded levels of HKB supplementation positively influenced broiler growth performance, as evidenced by significant improvements in body weight gain and feed efficiency. Moreover, HKB supplementation led to beneficial changes in cecal morphology, including increased villus height and villus to crypt ratio, indicating enhanced nutrient absorption and intestinal health. Additionally, HKB supplementation exerted regulatory effects on the cecal microbiota composition. Notably, specific bacterial genera such as *Thermoactinomyces*, *Allobaculum*, and *Bacillus*, as well as the fungal genus *Tausonia*, were consistently influenced by HKB supplementation across different dosage levels. While the exact interrelationships among these microbial components remain unclear, our study highlights the potential key players affected by HKB in the broiler gut. These results suggests that HKB supplementation holds promise as a dietary strategy to improve cecal morphology by modulating the composition of cecal bacteria and fungi, thereby enhancing overall growth performance. However, further investigations are warranted to unravel the intricate microbial interactions and the underlying mechanisms driving these effects.

## Conclusion

In conclusion, the addition of HKB to the diet improved the growth performance and cecal morphology of broilers. This may be related to the abundance changes of cecal *Thermoactinomyces*, *Allobaculum*, and *Bacillus*, as well as the fungal genus *Tausonia*. This study thus provides valuable insights into how HKB improve the growth of broilers.

## Data Availability

The data presented in the study are deposited in the figshare repository, accession number https://figshare.com/s/13ba334920a48aba5c6a.
