# Supplementary material for: Heat-killed Bacillus subtilis concerning broilers’ performance, cecal architecture and microbiota
Source: Front Microbiol. 2025 Jun 27;16:1606352. doi: 10.3389/fmicb.2025.1606352 (PMC12245841; doi:10.3389/fmicb.2025.1606352)
Supplement: Supplementary file 1 [file Table_1.docx]

Table 1 Composition and nutritional level of basal diet (air-dried basis)

| Ingredients, % | 1-21 days old | 22-42 days old |
| --- | --- | --- |
| Corn | 59.05 | 57.75 |
| Soya-bean oil | 2.00 | 6.00 |
| Corn gluten meal（60%） | 4.00 | 4.00 |
| Bean meal（45%） | 30.5 | 28.50 |
| Mountain flour | 1.40 | 1.00 |
| Calcium bicarbonate | 1.30 | 1.00 |
| Nacl | 0.25 | 0.25 |
| Premix | 1.50 | 1.50 |
| Total | 100 | 100 |
| Analyzed composition, % |  |  |
| Metabolizable energy, MJ/kg | 12.80 | 13.89 |
| Crude protein /% | 23.00 | 21.00 |
| Ca/% | 0.90 | 0.75 |
| total phosphorus /% | 0.60 | 0.50 |
| lysine /% | 1.415 | 1.308 |
| methionine /% | 0.563 | 0.456 |
| methionine + cystine /% | 0.971 | 0.881 |

^1^Provided per kg of complete diet: VA 10 000 IU, VD3 4 000 IU, VE 40 IU, VK 4 mg, VB_1_ 5 mg, VB_2_ 8 mg, niacin 65 mg, pantothenic acid 20 mg, VB_6_ 5 mg, biotin 0.5 mg, folic acid 2 mg, VB_12_ 0.02 mg, copper 9 mg, iron 85 mg, manganese 0.6 mg, iodine 0.6 mg.

^2^Nutrition levels are calculated values.
